# Three-Tesla Magnetic Resonance Imaging Characteristics of Hypertrophic Cardiomyopathy: A Comparison with Several Echocardiography Parameters

**DOI:** 10.31083/j.rcm2509341

**Published:** 2024-09-23

**Authors:** Phung Bao Ngoc, Vu Thi Kim Thoa, Vu Dang Luu, Pham Manh Hung, Nguyen Khoi Viet, Nguyen Ngoc Trang, Hoang Thi Van Hoa, Le Thi Thuy Lien, Nguyen Thi Huyen, Yung Liang Wan

**Affiliations:** ^1^Radiology Center, Bach Mai Hospital, 100000 Hanoi, Vietnam; ^2^Vietnam National Heart Institue, Bach Mai Hospital, 100000 Hanoi, Vietnam; ^3^Department of Medical Imaging and Intervention, Linkou Chang Gung Memorial Hospital, College of Medicine, Chang Gung University, 333 Taoyuan City, Taiwan

**Keywords:** hypertrophic cardiomyopathy, magnetic resonance imaging, echocardiography

## Abstract

**Background:**

Hypertrophic cardiomyopathy (HCM) is a primary cardiac disorder characterized by myocardial hypertrophy without increased afterload. This study set out to describe the cardiac magnetic resonance (CMR) imaging characteristics of HCM and to evaluate correlations of selected CMR parameters with echocardiography.

**Methods:**

This cross-sectional study enrolled 46 patients diagnosed at the Vietnam Heart Institute with HCM and underwent CMR at the Radiology Center, Bach Mai Hospital, from July 2021 to September 2022.

**Results:**

A left ventricular outflow tract (LVOT)/aortic valve (AO) diameter ratio of ≥0.38 on CMR was consistent with an LVOT pressure gradient (PG) of <30 mmHg on echocardiography. The LVOT diameter and the LVOT/AO diameter ratio differed significantly between obstructive and non-obstructive HCM. The predominant phenotypes were diffuse asymmetric HCM (32.6%) and septal HCM (37%), followed by apical HCM (6.5%). Most late gadolinium enhancement (LGE) lesions were observed in the mid-wall of the hypertrophic segments. The mean LGE mass was significantly higher in the obstructive group than in the non-obstructive HCM group (*p *< 0.05). A strong negative correlation (r = –0.66) was found between the LVOT/AO diameter ratio on the CMR and the LVOT PG via echocardiography. Moreover, echocardiography detected morphologic risk factors for sudden cardiac death (SCD) in 80.4% of patients, whereas the corresponding proportion detected by CMR was 91.3%. Patients with systolic anterior motion (SAM) had a risk for a LVOT/AO diameter ratio <0.38, which was 5.7 times the risk observed in their counterparts without SAM.

**Conclusions:**

The LVOT/AO diameter ratio detected by CMR is a precise index for classifying hemodynamic HCM groups. CMR was better than echocardiography for SCD risk stratification.

## 1. Introduction

Hypertrophic cardiomyopathy (HCM) is a primary cardiac disorder characterized by myocardial hypertrophy in the absence of any detectable increase in afterload (i.e., systemic hypertension or aortic stenosis). Still considered a disease burden worldwide, HCM has an estimated population prevalence of 0.2% (1/500) and is among the most common causes of sudden cardiac death (SCD), especially in patients under 35 years of age [1]. Therefore, the early and accurate diagnosis of HCM is crucial in the direction of care, timely treatment, and preventing complications.

HCM is divided into obstructive and non-obstructive groups based on peak gradient at the left ventricular outflow tract (LVOT) or mid-left ventricular cavity. LVOT obstruction is defined as a gradient greater than 30 mmHg that can lead to adverse outcomes. Usually, echocardiography is used to assess LVOT obstruction; however, echocardiography may not always be sufficient to diagnose HCM definitively, particularly in patients whose ultrasound window is limited and whose signs of hypertrophy are unclear [2]. In recent years, the emergence of cardiac magnetic resonance (CMR) imaging has overcome these echocardiography limitations and presents many obvious advantages, such as visualization of the myocardium and high reproducibility. CMR is now considered the reference standard for quantifying left ventricular (LV) volume, mass, wall thickness, function, and phenotypic classification. CMR has been recommended to aid echocardiography in definitive diagnosis, differential diagnosis, and treatment of HCM [3]. However, the correlation between the LVOT/aortic valve (AO) diameter ratio on CMR imaging and the LVOT pressure gradient (PG) on echocardiography has yet to be assessed adequately. In addition, HCM phenotypes vary among Europeans and Asians [4] and different countries within Asia [5, 6]. Therefore, we conducted this study to provide additional information on the imaging characteristics of HCM in Asians and evaluate the correlations of selected CMR parameters with echocardiography.

## 2. Materials and Methods

### 2.1 Patient Selection

Following the initial selection of 52 patients, 6 patients were excluded due to the non-diagnostic quality of CMR images. Finally, 46 patients diagnosed at the Vietnam National Heart Institute with HCM and underwent CMR at the Radiology Center, Bach Mai Hospital, from July 2021 to September 2022 were included.

#### 2.2.1 Selection Criteria

• The patient was diagnosed with HCM according to the European Society of Cardiology 2014 guidelines.

• The patient provided written informed consent to participate in this study.

#### 2.2.2 Exclusion Criteria

• The patient had a CMR contraindication (metal foreign body in the orbit, skull, heart, etc., or an implantable medical device such as a hearing aid or pacemaker).

• The patient had a medical, surgical, metabolic, or occupational condition that could be responsible for myocardial hypertrophy, such as hypertension or amyloidosis.

• The patient was allergic to contrast agents or had severe renal failure.

• The magnetic resonance images obtained were of non-diagnostic quality.

• The patient had claustrophobia or could not cooperate during CMR imaging.

### 2.2 CMR Imaging Protocols and Analysis

Images were acquired using a 3T magnetic resonance imaging machine (SIGNA Architect: GE HealthCare, Chicago, IL, USA). Heart MR images were obtained using a 30-channel adaptive image receive (AIR) anterior array coil and a 40-channel posterior array coil. Multiple-plane localizers were taken first, including axial, coronal, sagittal, two-chamber, three-chamber, four-chamber, short-axis, and LVOT views. Two-chamber, four-chamber, short-axis cine sequences (8–10 slices) from base to apex were obtained next. Then, 3-chamber and LVOT cine sequences were used to assess systolic anterior motion (SAM). One midventricular short-axis view for native T1 was acquired using modified look–locker inversion recovery, with 11 images and 17 heartbeats 3-(3)-3-(3)-5 balanced steady-state free precession sequences. The same short-axis view for T2 mapping was obtained using a T2 steady-state free precession sequence. Subsequently, late gadolinium enhancement (LGE) images with one slice on two-chamber and four-chamber views and eight slices on a short-axis view from base to apex were acquired 10 min after intravenous administration of gadolinium-based contrast (Dotarem: Guerbet, Villepinte, France) at a dose of 0.2 mmol/kg and a rate of 2–3 mL/s, followed by administration of 20–25 mL saline. Finally, postcontrast modified look–locker inversion recovery T1 mapping was obtained on the same short-axis slice previously used for the precontrast T1 mapping. Two radiologists with over five years of experience analyzed the data using MR Workspace (Philips Medical Systems, Eindhoven, The Netherlands) with CVi42 software (Circle Cardiovascular Imaging, Calgary, AB, Canada). LV mass, ejection fraction (EF), LV end-systolic volume, end-diastolic volume, wall thickness, and LVOT diameter were calculated. Consensus was obtained following a discussion of any discrepancies between the two radiologists.

### 2.3 Statistical Analysis

Data are presented as the mean ± standard deviation. Spearman correlation coefficients were used to assess associations. A *t-*test was used to analyze categorical variables. To determine the cut-off for the LVOT/AO diameter ratio in the diagnosis of obstructive HCM, a receiver operating characteristic curve was generated. Data were processed using IBM SPSS Statistics for Windows (version 20.0: IBM Corporation, Armonk, NY, USA). Statistical significance was assumed at a *p*-value less than 0.05.

## 3. Results

### 3.1 Clinical Features of the Study Population

The 46 study patients comprised 27 men (58.7%) and 19 women (41.3%). The mean age in the cohort was 51.2 ± 18.4 years, ranging from 17 to 84 years. The mean body mass index was 22.6 ± 3.4 kg/m^2^, and the average heart rate was 80.2 ± 21.0 bpm.

Concerning clinical characteristics, 15 patients (32.5%) had a family history of HCM, 14 (30.4%) had a family history of SCD, 1 (2.2%) had a family history of implantable cardioverter–defibrillator, 34 (73.9%) had dyspnea, and 36 (78.3%) had chest pain. The echocardiogram features and phenotypic findings for HCM are shown in **Supplementary Tables 1,2**, respectively.

### 3.2 HCM Imaging Characteristics

**Fig. 1.**
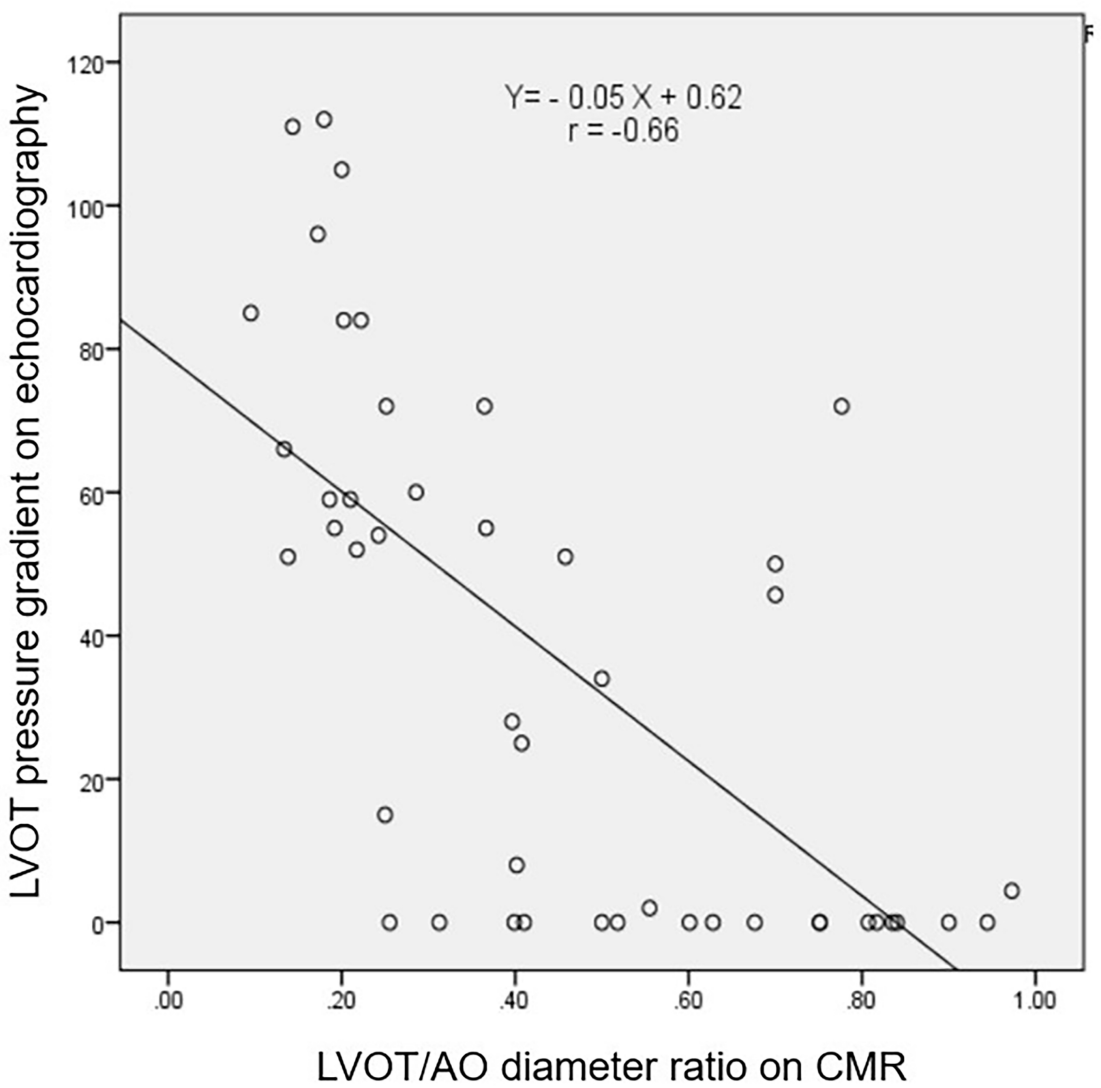
**Correlation between the LVOT PG on echocardiograph and the LVOT/AO diameter ratio on cardiac magnetic resonance image**. The figure shows a strong negative correlation (r = –0.66) between the LVOT PG on echocardiography and the LVOT/AO diameter ratio on cardiac magnetic resonance imaging. LVOT, left ventricular outflow tract; AO, aortic; PG, pressure gradient; CMR, cardiac magnetic resonance.

**Fig. 2.**
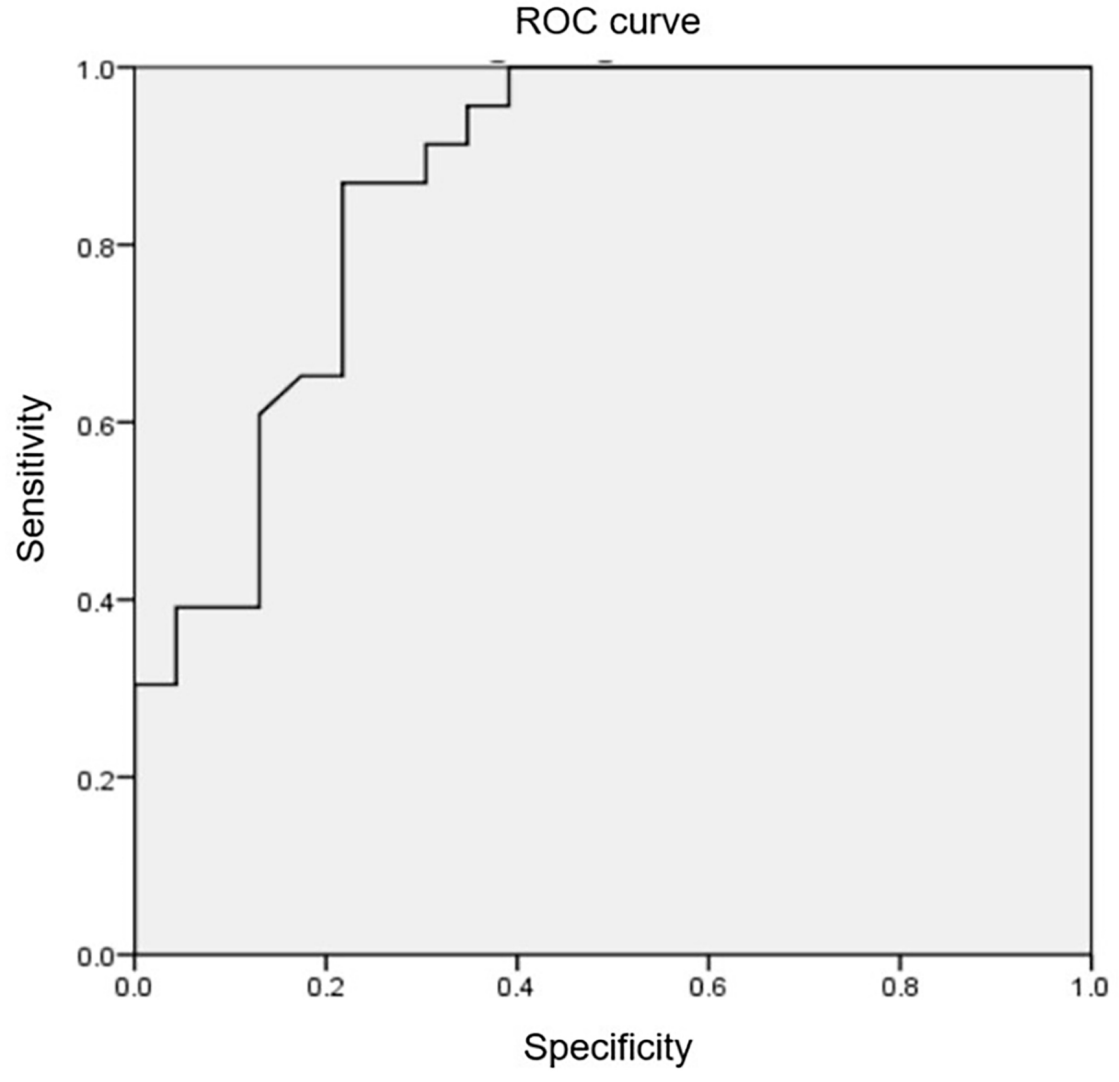
**The receiver operating characteristic curve and the cut-off point of LVOT/AO diameter ratio**. Utilizing the receiver operating characteristic curve, the LVOT/AO diameter ratio on cardiac magnetic resonance imaging was identified as 0.38, with an area under the curve of 0.87, a sensitivity of 87%, and a specificity of 78.3%, allowing for the detection of an LVOT PG <30 mmHg at rest in HCM patients. LVOT, left ventricular outflow tract; AO, aortic; PG, pressure gradient; HCM, hypertrophic cardiomyopathy; ROC, receiver operating characteristic.

HCM was classified as obstructive or non-obstructive on CMR based on comparing the LVOT/AO diameter ratio with the LVOT PG on echocardiography, using a receiver operating characteristic curve to determine the cut-off point (Figs. 1,2). Of the 46 patients, 21 (45.7%) had obstructive HCM based on the LVOT/AO diameter ratio.

The assessment of LVOT/AO diameter ratio on the 3-chamber steady-state free precession CMR image, cine 4-chamber view obtained at end-diastolic phase, the severe LVOT obstruction with positive SAM on cine 3-chamber and LVOT views, and late gadolinium enhancement with a transmural and mid-wall pattern are shown on **Supplementary Figs. 1–4**, respectively.

Table 1 compares the LV parameters on the CMR between the obstructive and non-obstructive HCM groups. No significant differences were observed between the groups (*p* > 0.05) concerning end-diastolic volume, end-systolic volume, ejection fraction, LV mass, maximum wall thickness, and left atrial volume index; however, a significant difference (*p* < 0.05) was observed for the LVOT diameter, LVOT/AO diameter ratio, and native T1 value.

**Table 1.** **LV parameters of the obstructive and non-obstructive hypertrophic cardiomyopathy on cardiac magnetic resonance**.

Parameters	Obstructive HCM (n = 21, $\bar{x}$ ± SD)	Non-obstructive HCM (n = 25, $\bar{x}$ ± SD)	*p*-value
LV EDV (mL)	110.7 ± 30.3	117.3 ± 29.69	0.46
LV ESV (mL)	34.2 ± 12.64	42.95 ± 22.28	0.117
LV EF (%)	69.03 ± 8.13	64.3 ± 11.36	0.119
LV mass (g)	165.3 ± 59.9	182.3 ± 60.49	0.332
Maximum wall thickness (mm)	22.4 ± 5.45	22.8 ± 2.25	0.805
LVAi (mL/m^2^)	53.4 ± 24.48	41.2 ± 21.08	0.09
LVOT diameter (mm)	4.5 ± 1.62	13.3 ± 3.72	0.00
LVOT/AO diameter ratio	0.22 ± 0.07	0.65 ± 0.19	0.00
Native T1 (ms)	1259 ± 67.5	1198 ± 98.3	0.014

LV, left ventricular; EDV, end-diastolic volume; ESV, end-systolic volume; EF, ejection fraction; LAVi, left atrial volume index; LVOT, left ventricular outflow tract; AO, aortic; HCM, hypertrophic cardiomyopathy.

Table 2 presents the HCM phenotypes for the CMR. Of the 46 patients, 3 (6.5%) had bilateral ventricle hypertrophy, and 4 (8.7%) had concentric HCM. Septal HCM accounted for the highest proportion of cases (37%, 17/46), followed by diffuse symmetric HCM (32.6%, 15/46). Notably, the proportion of the septal phenotype in the two groups was significantly different (*p* = 0.009).

**Table 2.** **Hypertrophic cardiomyopathy phenotypes on the cardiac magnetic resonance imaging**.

HCM phenotypes on the cardiac magnetic resonance imaging			Obstructive (n = 21)		Non-obstructive (n = 25)		Total (n = 46)		*p-*value
			n	%	n	%	n	%	
LV (n = 43)	Asymmetric HCM (n = 39)	Diffuse asymmetric HCM	5	23.8	10	40.0	15	32.6	0.24
		Septal HCM	12	57.1	5	20.0	17	37.0	0.009
		Mid septal HCM	0	0	3	12.0	3	6.5	0.24
		Apical septal HCM	1	4.8	0	0	1	2.2	0.5
		Apical HCM	2	9.5	1	4.0	3	6.5	0.6
	Concentric HCM		0	0	4	16.0	4	8.7	0.1
Both left and right ventricles			1	4.8	2	8.0	3	6.5	0.7

HCM, hypertrophic cardiomyopathy; LV, left ventricular.

LGE was observed via CMR imaging for 23 patients (Table 3). The mean LGE mass in the obstructive group was higher than in the non-obstructive group (20.6 ± 23.6 vs. 10.8 ± 13.0, *p* = 0.04). Most LGE lesions appeared in hypertrophic segments (87%) and the mid-wall (69.6%), with patchy enhancement (82.6%).

**Table 3.** **Late gadolinium enhancement patterns on the cardiac magnetic resonance imaging**.

LGE (n = 23)		Obstructive (n = 9)		Non-obstructive (n = 14)		Total (n = 23)		*p*-value
		n	%	n	%	n	%	
Hypertrophic segment		8	88.9	12	85.7	20	87.0	0.6
Distribution	Subendocardial	2	22.2	0	0	2	8.7	0.08
	Epicardial	2	22.2	4	28.6	6	26.1	0.4
	Mid-wall	6	66.7	10	71.4	16	69.6	0.5
	Transmural	1	11.1	4	28.6	5	21.7	0.3
Patterns	Nodule	3	33.3	4	28.6	7	30.4	0.8
	Patchy	7	77.8	12	85.7	19	82.6	0.7
	Linear	5	55.6	4	28.6	9	39.1	0.2
LGE mass (g/m^2^)		20.6 ± 23.6		10.8 ± 13.0		14.6 ± 18.01		0.04

LGE, late gadolinium enhancement.

Table 4 shows that 52.2% of patients had SAM, and 10.9% had ventricular aneurysms. The SAM was significantly more common in obstructive HCM than in non-obstructive HCM (90.5% vs. 20.0%, *p* = 0.00).

**Table 4.** **Systolic anterior motion and ventricular aneurysm features of cardiac magnetic resonance imaging**.

	Obstructive (n = 21)		Non-obstructive (n = 25)		Total (n = 46)		*p-*value
	n	%	n	%	n	%	
Systolic anterior motion	19	90.5	5	20.0	24	52.2	0.00
Ventricular aneurysm	1	4.0	4	19.0	5	10.9	0.1

### 3.3 Correlation between Several Parameters on CMR and Echocardiography

Table 5 indicates that CMR was better for evaluating maximum wall thickness ≥30 mm and EF <50% and stratifying SCD risk than echocardiography. Only CMR imaging could detect ventricular aneurysms and LGE; echocardiography and CMR had similar detection rates for SAM.

**Table 5.** **Agreement between echocardiography and CMR in HCM**.

	Echocardiography (n, %)	CMR (n, %)	Kappa value
Wall thickness ≥30 mm	3 (6.5)	6 (13.0)	0.3
Ventricular aneurysm	-	5 (10.9)	-
LGE	-	23 (50.0)	-
EF <50%	2 (4.3)	4 (8.7)	0.3
SCD risk	37 (80.4)	42 (91.3)	0.2
SAM	25 (54.3)	24 (52.2)	0.6

HCM, hypertrophic cardiomyopathy; CMR, cardiac magnetic resonance; LGE, late gadolinium enhancement; EF, ejection fraction; SCD, sudden cardiac death; SAM, systolic anterior motion.

**Supplementary Table 3** showed that patients with SAM exhibited a 5.7 times higher risk of having an LVOT/AO diameter ratio <0.38 than those without SAM (*p* < 0.05).

## 4. Discussion

In this cohort, the 46 patients diagnosed with HCM had an average age of 51.2 ± 18.43 years. The male-to-female ratio was 1:4, which was consistent with the study by Corona-Villalobos *et al*. [7]. HCM is caused primarily by an autosomal dominant mutation that can be passed to the next generation; the risk is 50% if either parent has HCM [8]. In this study, 32.5% of the patients had a family history of HCM, 30.4% had a family history of SCD, and 2.2% had a family history of implantable cardioverter–defibrillator use, rates that were notably higher than those reported by Chan *et al*. [9], who found rates of 13.3%, 3.9%, and 2.2% respectively. The rates reported in this study were also much higher than those reported by Alashi *et al*. [10], who found a family history of HCM and SCD in 2% and 5% of cases, respectively. These differences could be attributable to variations in sample size, study subjects, and research locations. In particular, Alashi *et al*. [10] recruited 1110 older patients aged 75–92 years (mean: 80 ± 5 years) in the United States. Chan *et al*. [9] recruited 564 Chinese children with a median age at diagnosis of 1.0 years.

In this study, patients experienced syncope, dyspnea, and chest pain as their most common symptoms. The prevalence of dyspnea, at 73.9% (approaching New York Heart Association class II), was comparable to findings reported by Alashi *et al*. [10]. However, the rates of chest pain and syncope in the current study (78.3% and 23.9%, respectively) were higher than the 19% and 12% reported in the study by Alashi *et al*. [10], although the variations in age distribution and a higher proportion of patients with heart failure in the current study might have influenced these differences.

Our study revealed a strong negative correlation between LVOT PG on echocardiography and the LVOT/AO diameter ratio on CMR (r = –0.66). Using a receiver operating characteristic curve, we established that an LVOT/AO diameter ratio of 0.38 measured by CMR, with an area under the curve of 0.87, a sensitivity of 87%, and a specificity of 78.3%, allowed for the detection of an LVOT PG <30 mmHg in patients with HCM at rest. To our knowledge, this is one of the few studies that determine a LVOT/AO diameter ratio cut-off value for CMR in assessing LVOT obstruction. In comparison, Vogel-Claussen *et al*. [11] studied 92 patients and identified an LVOT/AO diameter ratio cut-off point of 0.33, with an area under the curve of 0.91, a sensitivity of 91%, and a specificity of 80%. This variance in cut-off points could be attributable to differences in sample size and patient characteristics.

The mean maximum LV wall thickness observed in this study was similar to the previously reported value of 22 ± 5 mm [12]. The mid-anteroseptal, mid-inferoseptal, and basal anterior segments had the highest mean thicknesses, whereas the basal inferior segment had the smallest thickness, consistent with values reported by Maron *et al*. [12]. Bilateral ventricular hypertrophy was present in 6.5% of the patients in this study. The predominant phenotypes were diffuse asymmetric HCM and septal HCM, followed by apical HCM at 6.5%. This ranking aligns with the findings of Thao *et al*. [13], who observed asymmetric hypertrophy (40.7%), diffuse hypertrophy (44.5%), apical HCM (14.8%), and bilateral ventricular hypertrophy (18.5%) in 27 patients with HCM. Compared with a study of 25 patients with HCM by Kim *et al*. [14], this study found a similar predominance of diffuse asymmetric HCM (32%) and septal HCM (52%), with a slightly higher prevalence of apical HCM (16%). The proportion of apical hypertrophic cardiomyopathy of 6.5% in our study was lower than those reported in Asia (31%) and Europe (13%) [4]. On the contrary, the proportion of septal hypertrophic cardiomyopathy in this study (37%) was higher than those in Asia and Europe (17% and 28%, respectively) [4]. This difference may be partly attributed to regional variations and the small sample size in the study.

An outstanding advantage of CMR imaging compared with echocardiography is the detection of myocardial fibrosis by LGE, not only in hypertrophic segments but also in non-hypertrophic areas. LGE mass plays a role in disease progression and prognosis and is possibly the cause of ventricular arrhythmias. Half of the study patients had LGE, a proportion lower than that reported by Thao *et al*. [13] (88.9%), likely because of differences in sample size, study location, time, and disease stage. Some studies have revealed that the rate of LGE varies and can be as high as 60%–70% in adults and 46% in children [13, 15]. These results are similar to those reported by Mentias *et al*. [16]. The distribution of LGE mainly in the mid-wall of the hypertrophic segments with plaque pattern in the current study accords with findings in a previous series [17]. These features are consistent with the characteristics of nonischemic cardiomyopathy and do not fall into coronary artery territory. The LGE measurement indicated the amount of fibrous tissue, denoting disease progression and prognosis. In this study, the mean LGE mass in the obstructive HCM group was twice that in the non-obstructive HCM group. In addition, native T1 values could reflect histologic remodeling of the myocardium and become widely used in clinical practice [18]. In this study, the native T1 value was higher in the obstructive HCM group than in the non-obstructive HCM group, denoting more advanced myocardial tissue remodeling in the obstructive group.

In cardiovascular disease, echocardiography is a non-invasive, widely available, and valuable diagnostic tool, whereas CMR is a high-cost test that can complement and address the limitations of echocardiography. A LV wall thickness ≥30 mm represents a prognostic factor independently associated with SCD. Therefore, accurate wall thickness assessment is pivotal in diagnostic and therapeutic decision-making, such as for pacemaker implantation. In a study that compared maximum wall thickness measured by echocardiography and CMR, the discrepancy ranged from 3 mm to 17 mm, indicating that methodology differences could affect treatment decisions [19]. Echocardiography has been reported to potentially either overestimate or underestimate HCM because of the challenges in obtaining optimal images for all LV areas [19, 20]. Since measurement discrepancies can influence diagnosis and treatment, CMR is increasingly considered a routine investigation for all patients with HCM, particularly when the ultrasound window is limited. In this study, we observed no differences in the EF values obtained using the two methods. This finding contrasts with a study by Jenkins *et al*. [21], in which EF measured by CMR and 3 dimension (3D) echocardiography demonstrated accuracy superior to that obtained with 2 dimension (2D) echocardiography. The discrepancy might stem from differences in patient populations.

The 2020 American College of Cardiology/American Heart Association guideline outlines seven risk factors for HCM SCD risk stratification, including a family history of sudden death from HCM, wall thickness ≥30 mm, unexplained syncope, LV apical aneurysm, extensive LGE on CMR imaging, and non-sustained ventricular tachycardia diagnosed by ambulatory monitor. In this study, echocardiography detected 80.4% of patients at risk of SCD; CMR could identify up to 91.3%, necessitating consideration for pacemaker placement. This observation underscores the superiority of CMR to echocardiography. Further, with its high resolution and ability to delineate fibrous tissue, CMR provides more accurate information. However, in clinical practice, ideal prognostic parameters should be simple and readily available, highlighting structural anomalies such as myocardial fibrosis and systolic and diastolic dysfunction. We therefore aver that an effective SCD screening strategy combines echocardiography and CMR. In this study, 8.7% of patients had extensive LGE (≥15% of LV mass), independently predicting SCD risk in patients with HCM. In a study that followed 1293 patients with HCM for over 3.3 years, every 10% increase in LGE mass was associated with a 1.46 increased risk of SCD [22], underscoring the prognostic significance of LGE.

This study further identified a negative correlation between the LVOT/AO diameter ratio according to CMR and the LVOT PG according to echocardiography, consistent with previous reports [11, 23]. SAM of the mitral valve narrows the LVOT, reducing the LV ejection volume. As a compensatory mechanism, the heart contracts rapidly and forcefully, elevating the velocity through the LVOT and resulting in increased LVOT PG. In this study, without examining LVOT PG directly, we found that those with SAM had a 5.7 times greater likelihood of having an LVOT/AO diameter ratio <0.38 than those without SAM. To our knowledge, this is the first study on SAM of the mitral valve on echocardiography to predict LVOT/AO diameter ratio on CMR in patients with HCM.

### Limitations

This study has certain limitations. First, it was conducted at a single center with a relatively small sample size. Second, genetic testing was only performed on some patients. Finally, the follow-up duration was short; only seven patients underwent myomectomy. Subsequently, further research is needed to address these issues.

## 5. Conclusions

The LVOT/AO diameter ratio measured by CMR represents a precise index for categorizing hemodynamic HCM groups. The findings of this study suggest that CMR outperforms echocardiography in the stratification of SCD risk.

## Availability of Data and Materials

The datasets analyzed during this study are available from the corresponding author upon reasonable request.

## Abbreviations

HCM, hypertrophic cardiomyopathy; CMR, cardiac magnetic resonance; LV, left ventricular; LVOT, LV outflow tract; AO, aortic valve; PG, pressure gradient; LGE, late gadolinium enhancement; SCD, sudden cardiac death; SAM, systolic anterior motion.

## Supplementary Material

Supplementary material associated with this article can be found, in the online version, at https://doi.org/10.31083/j.rcm2509341.
