# Supplementary material for: Three-Tesla Magnetic Resonance Imaging Characteristics of Hypertrophic Cardiomyopathy: A Comparison with Several Echocardiography Parameters
Source: Rev Cardiovasc Med. 2024 Sep 23;25(9):341. doi: 10.31083/j.rcm2509341 (PMC11440385; doi:10.31083/j.rcm2509341)
Supplement: Supplementary file 1 [file 2153-8174-25-9-341-s1.docx]

**Supplementary Tables and Figures**

**Supplementary Table 1. Hypertrophic cardiomyopathy features on** **echocardiographs (n = 46).**

| **Parameters** | **Mean ± SD** | **Maximum** | **Minimum** |
| --- | --- | --- | --- |
| Maximal-end-diastolic-thickness of LV (mm) | 18.60 ± 4.98 | 33 | 13 |
| Dd (mm) | 41.90 ± 6.91 | 52 | 31 |
| Ds (mm) | 25.60 ± 4.88 | 39 | 17 |
| EDV (ml) | 81.20 ± 23.96 | 132 | 40 |
| ESV (ml) | 25.40 ± 12.14 | 66 | 8 |
| EF (%) | 68.50 ± 9.05 | 85 | 42 |
| IVSd (mm) | 15.50 ± 5.59 | 33 | 6 |
| IVSs (mm) | 18.40 ± 5.95 | 38 | 10 |
| Left atrial diameter (mm) | 37.02 ± 6.91 | 53 | 26 |
| Ascending aortic diameter (mm) | 30.60 ± 3.56 | 37 | 23 |

LV, left ventricular; D, dimension; EDV, end-diastolic volume; ESV, end-systolic volume; EF, ejection fraction; IVS, interventricular septum; -d, in diastole; -s, in systole.

**Supplementary Table 2. Hypertrophic cardiomyopathy phenotypes on echocardiographs.**

| **HCM phenotypes** | | **Patients**  **(n = 46)** | **Percentages (%)** |
| --- | --- | --- | --- |
| Left ventricle | Diffuse HCM | 2 | 4.3 |
|  | Septal HCM | 37 | 80.5 |
|  | Apical HCM | 1 | 2.2 |
|  | Concentric HCM | 4 | 8.8 |
| Both left and right ventricles | | 2 | 4.3 |

HCM, hypertrophic cardiomyopathy.

**Supplementary Table 3. The risk of having an LVOT/Ao diameter ratio < 0.38 in patients without and with SAM**

| **SAM** | **LVOT/Ao diameter ratio < 0.38** | **OR (95 % CI)** |
| --- | --- | --- |
| (-) | 9.1 % | 1 |
| (+) | 79.2 % | 5.7 (1.6; 12.7) |

LVOT left ventricular outflow tract; Ao, aortic; CMR, cardiac magnetic resonance; SAM, systolic anterior motion; OR, odds ratio; CI, confidence interval.

**
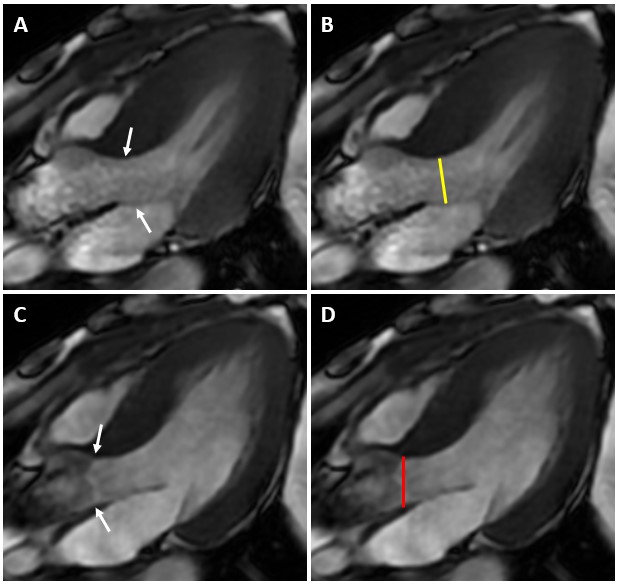
**

**Supplementary Fig. 1. Assessment of LVOT/Ao diameter ratio on 3-chamber steady-state free precession cardiac magnetic resonance image.** The LVOT diameter is identified as the minimal distance between the interventricular septum and the anterior leaflet of the mitral valve during systole (A (arrows), B (yellow line)). The aortic diameter is measured at the aortic annulus in the end-diastole (C (arrows), D (red line)). The LVOT diameter was divided by the aortic diameter to calculate the LVOT/Ao diameter ratio. LVOT, left ventricular outflow tract; Ao, aortic.


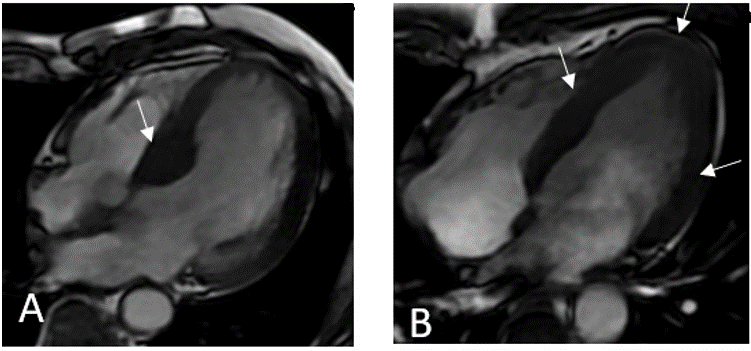


**Supplementary Fig. 2. Cine 4-chamber view obtained at end-diastolic phase.** (A) Cardiac magnetic resonance image of a 70-year-old male shows midseptal hypertrophic cardiomyopathy (arrow); (B) cardiac magnetic resonance image of a 46-year-old male shows concentric hypertrophic cardiomyopathy (arrows).


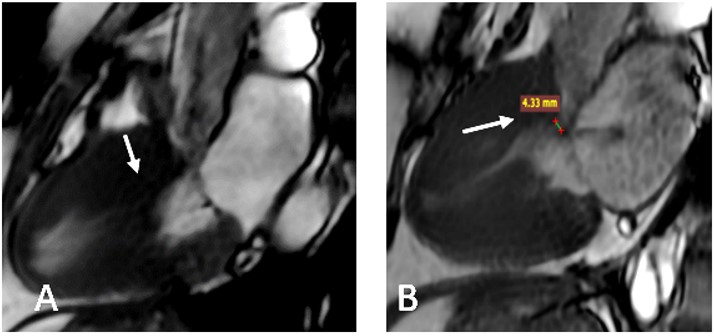


**Supplementary Fig. 3.** A 72-year-old female had severe LVOT obstruction with positive SAM on cine 3-chamber view (A) and LVOT view (B). SAM, systolic anterior motion; LVOT, left ventricular outflow tract.

*
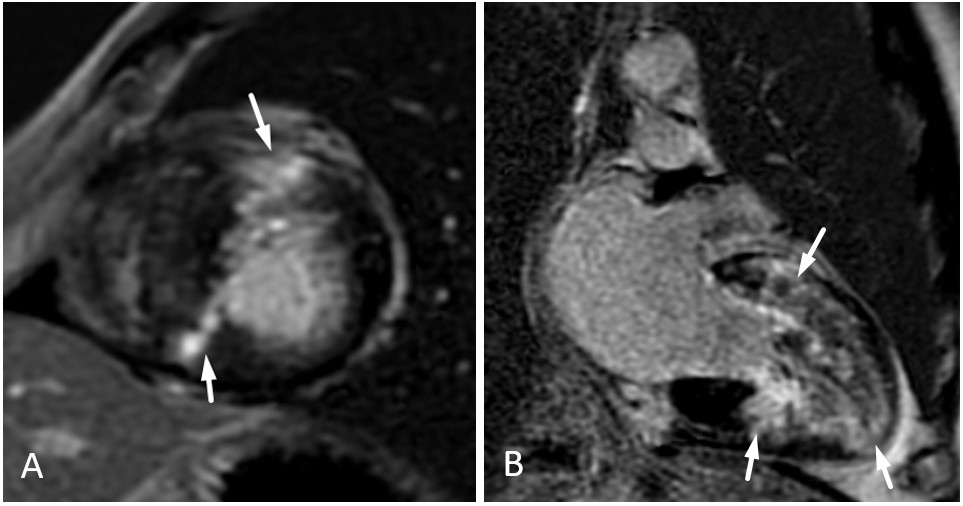
*

**Supplementary Fig. 4.** A 55-year-old male had late gadolinium enhancement (arrows) with a transmural pattern on a short-axis view (A) and a midwall pattern on a 2-chamber view (B).
